# The nose–brain axis as a neuroimmune conduit: how allergic rhinitis rewires the central nervous system

**DOI:** 10.3389/fimmu.2026.1920083

**Published:** 2026-10-02

**Authors:** Zhuoqin Han, Fengli Cheng, Yanjie Wang, Haoxiang Zhang, Luyao Wang, Changqing Zhao

**Affiliations:** 1 Department of Otolaryngology-Head and Neck Surgery, The Second Hospital of Shanxi Medical University, Taiyuan, Shanxi, China; 2 Shanxi Key Laboratory of Rapid Diagnosis and Precision Treatment of Airway Allergic Diseases, Taiyuan, Shanxi, China

**Keywords:** allergic rhinitis, astrocytes, central sensitization, microglia, neuroimmune crosstalk, nose-brain axis, trigeminal nerve

## Abstract

Allergic rhinitis (AR) has traditionally been regarded as a type I hypersensitivity disorder confined to the nasal mucosa. However, persistent symptoms, nasal hyperresponsiveness, and comorbid neuropsychiatric disturbances—including headache, fatigue, cognitive impairment (“brain fog”), anxiety, and depression—cannot be fully explained by peripheral inflammation alone, thereby implicating the involvement of central sensitization. The nose-brain axis serves as a critical bidirectional conduit that relays peripheral inflammatory signals to the central nervous system (CNS) and initiates neuroimmune crosstalk, which is now recognized as a core driver of central sensitization in AR. In this review, we systematically delineate the hierarchical mechanisms by which the nose-brain axis mediates central sensitization in AR, encompassing: peripheral neuroimmune synapse formation, trigeminal afferent signaling, central integration within the brainstem and thalamus, functional remodeling of limbic and sensory cortical regions, microglial and astrocytic activation, central cytokine network dysregulation, glutamatergic hyperexcitation, and descending modulatory imbalance. We further summarize emerging therapeutic strategies targeting the nose-brain axis, including peripheral nerve modulation, suppression of central neuroinflammation, restoration of neurotransmitter homeostasis, and psychobehavioral interventions. This review provides a unified mechanistic framework for understanding neuroinflammation and central sensitization in AR and offers novel insights for developing targeted therapies for patients with refractory AR and central comorbidities.

## 1 Introduction

Allergic rhinitis (AR) is a highly prevalent chronic inflammatory disease with a worldwide distribution. Traditionally viewed as a type I hypersensitivity disorder initiated by allergen exposure, AR is characterized by Th2-polarized immune responses and nasal mucosal inflammation (1, 2). Nevertheless, a substantial proportion of patients continue to experience persistent nasal hyperresponsiveness, ongoing symptoms (e.g., pruritus and rhinorrhea), and CNS-related disturbances—including headache, fatigue, cognitive dysfunction, and mood disorders—even when allergen exposure is minimized or local inflammation is adequately controlled (3, 4). These phenomena cannot be fully accounted for by peripheral inflammation alone, suggesting the propagation of pathological signals from the periphery to the CNS and subsequent functional remodeling of central circuits—a process collectively termed central sensitization (4, 5).

In recent years, the concept of the “nose-brain axis” has emerged as an integrative framework describing bidirectional communication between the nasal cavity and the brain via neural, immune, and endocrine pathways (6, 7). Aberrant activation of this axis is increasingly recognized as a key driver of central sensitization in AR (7, 8). In this review, we systematically examine the multi-level mechanisms through which the nose-brain axis contributes to central sensitization in AR and highlight future directions for translational investigation.

## 2 Concept and anatomophysiological basis of the nose-brain axis

The nose-brain axis constitutes a bidirectional communication network that integrates immune and sensory signals originating in the nasal cavity and transmits them to the central nervous system (CNS), while also receiving regulatory input from central structures (6). A diverse array of substances—including odorants, pathogens, inflammatory mediators, and exogenous agents—can access the cranial cavity via the olfactory nerve, trigeminal nerve, or humoral routes, thereby facilitating signal transmission from the periphery to the CNS (5).

This axis comprises three principal components: the peripheral compartment (nasal mucosal epithelium, resident immune cells, and trigeminal sensory nerve endings), the conducting pathways (trigeminal, olfactory nerves and humoral pathway), and the central compartment (trigeminal spinal nucleus, nucleus tractus solitarius, thalamus, limbic system, and prefrontal cortex) (6, 9, 10). Through the nose-brain axis, local nasal inflammation can activate CNS-resident microglia and astrocytes, triggering neuroinflammation that contributes to the pathogenesis of CNS disorders (3, 5) (**Figure 1**).

**Figure 1:**
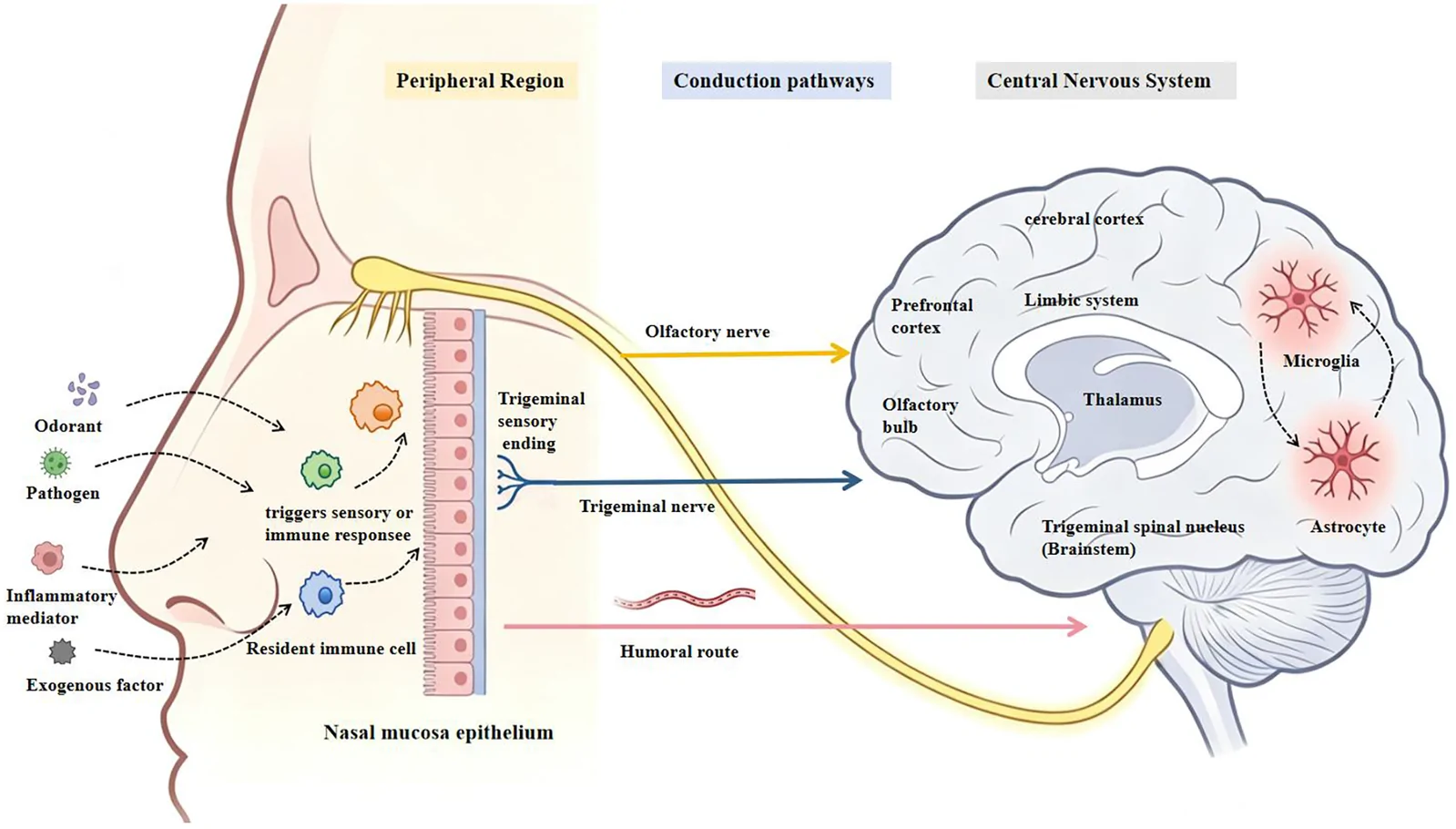
Schematic representation of the nose-brain axis mediating neuroimmune crosstalk and central sensitization in allergic rhinitis. This figure provides the macroscopic anatomical and cellular roadmap of the nose–brain axis. Nasal stimuli (odorants, pathogens, inflammatory mediators, microbiota) trigger signaling to the central nervous system via olfactory, trigeminal, and humoral routes. Peripheral activation of nasal neurons and immune cells propagates signals to the olfactory bulb, brainstem, thalamus, amygdala, and higher cortical regions, culminating in glial activation (microglia, astrocytes) and central neuroinflammatory cascades. This overarching framework serves as the top-level guide for the subsequent mechanistic details presented in **Figures 2** and **3**.

**Figure 2:**
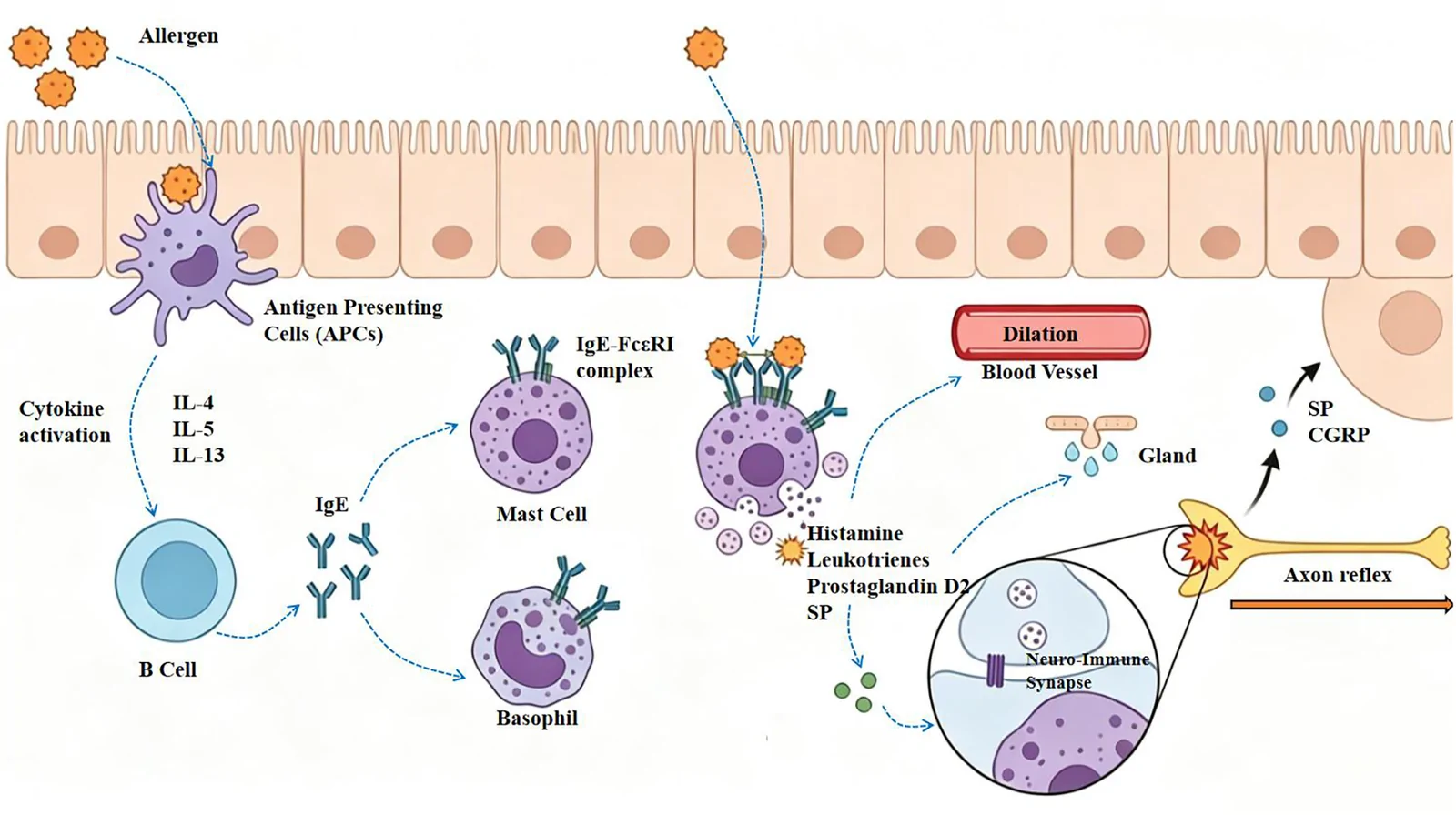
Mechanisms of allergic rhinitis: the nasal immune response and neuro-immune synapse. Inhaled allergens captured by nasal APCs trigger a Th2 response, releasing IL-4, IL-5, and IL-13 that drive B cells to produce allergen-specific IgE. IgE binds to FcϵRI on mast cells and basophils, inducing degranulation and release of histamine, leukotrienes, prostaglandin, bradykinin, and other mediators. These mediators cause vasodilation, glandular secretion, and activate sensory nerves, which then release SP and calcitonin gene-related peptide (CGRP) via axon reflex. SP acts on NK1 receptors, and CGRP on CGRP receptors (CGRPR); both are expressed on nasal vascular endothelium, smooth muscle, glands, mast cells, and eosinophils. At neuro-immune synapses, nerve-derived SP and CGRP act on adjacent mast cells or dendritic cells, rapidly modulating local mediator release. This neuro-immune crosstalk amplifies inflammation and perpetuates allergic symptoms.

**Figure 3:**
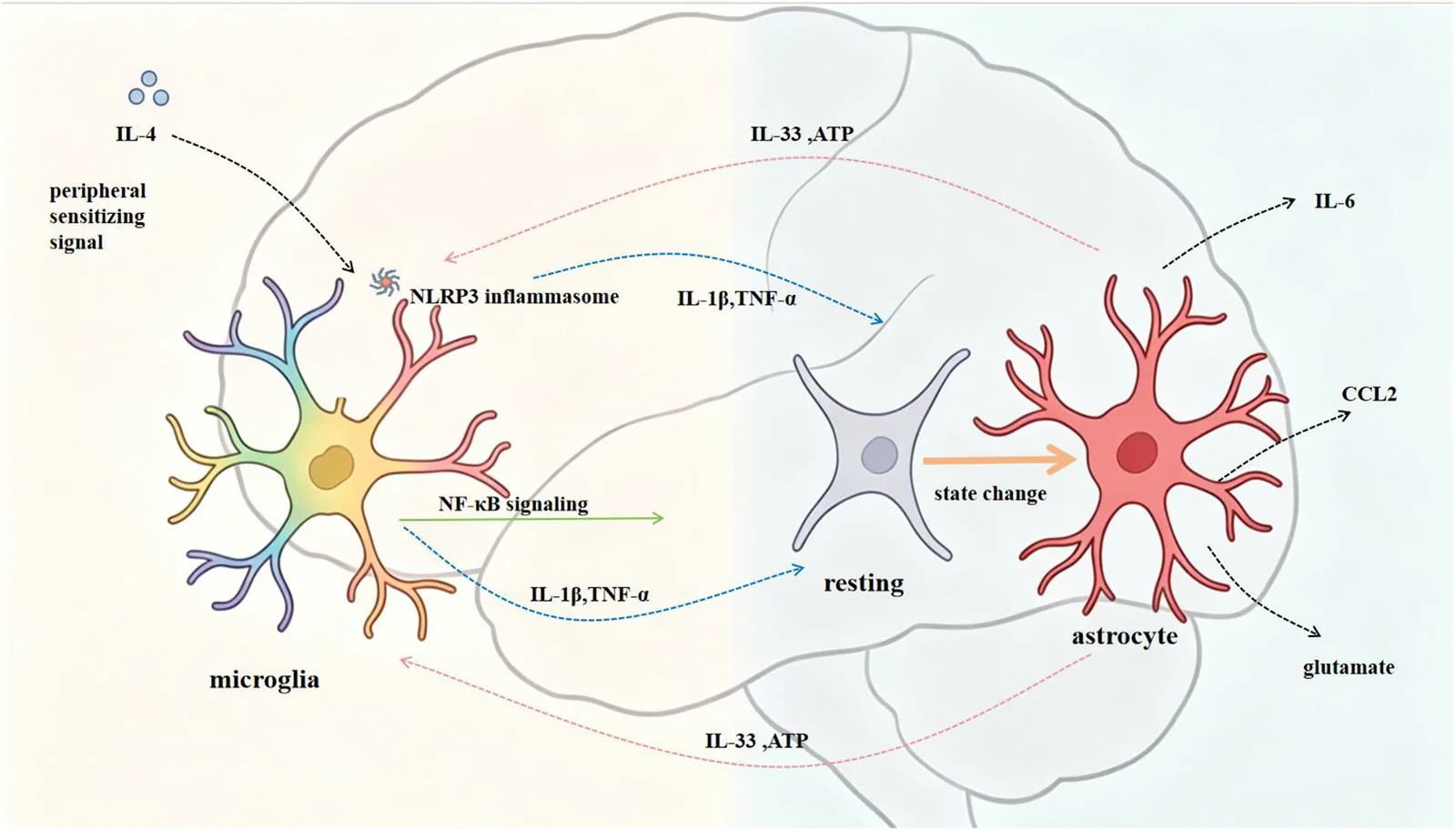
Synergistic amplification mechanism between microglia and astrocytes in central sensitization of allergic rhinitis. Peripheral signals (e.g., IL-4) activate microglia, which release IL-1β and TNF-α via the NLRP3 inflammasome and NF-κB pathways. These cytokines convert resting astrocytes into a reactive state (A1 phenotype), leading to production of IL-6, CCL2, and glutamate. Reactive astrocytes in turn sustain microglial activation through IL-33 and ATP, forming a bidirectional positive feedback loop. The resulting IL-1β-enriched milieu upregulates neuronal excitatory receptors (e.g., NMDA receptors) and reduces inhibitory tone, thereby inducing and maintaining central sensitization in allergic rhinitis.

## 3 Peripheral initiation: how AR inflammation activates and sensitizes primary afferent nerves

### 3.1 Formation of the neuroimmune synapse in the nasal mucosa

AR is fundamentally an IgE-mediated type 2 inflammatory response triggered by allergen exposure (11, 12). Allergens cross-link high-affinity FcϵRI receptors on mast cells, inducing degranulation and the release of histamine, leukotrienes, and proteases (13, 14). Beyond their classical roles, these mediators function as critical messengers that initiate neural signal transduction (15, 16). They directly activate receptors on sensory nerve endings—including the histamine H1 receptor (H1R), protease-activated receptor 2 (PAR2), prostaglandin E receptors (EP), and cysteinyl leukotriene receptor 1 (CysLT1R)—thereby generating action potentials that propagate centrally (17, 18). This direct activation constitutes the initial step of neurogenic inflammation, converting local chemical signals into electrical impulses (19, 20).

Within the nasal mucosa, sensory nerve endings and immune cells are spatially interwoven, forming a highly coordinated functional unit termed the “neuroimmune synapse” (21, 22).This synapse acts as an active bidirectional communication hub: on the one hand, it rapidly conveys immune information to the nervous system, providing initial signals for central sensitization (4, 23); on the other hand, neurotransmitters released from nerve endings reciprocally amplify local inflammatory responses (15, 18). This dynamic interplay indicates that, in AR, the immune and nervous systems function as an integrated entity (**Figure 2**).

### 3.2 Sensitization of sensory neurons and neuropeptide release

Persistent exposure to inflammatory mediators is the primary driver of peripheral sensitization. These mediators act continuously on trigeminal nerve endings, particularly on nociceptive neurons expressing TRPV1 and TRPA1 channels (24, 25). TRPV1 channels are sensitized by a variety of endogenous inflammatory mediators and second messengers, positioning them as pivotal determinants of nociceptive signaling (18, 26). In allergic rhinitis (AR), inflammatory mediators induce TRPV1 phosphorylation via PKA- and PKC-dependent pathways, altering channel gating properties and lowering activation thresholds (27, 28). Animal models of AR exhibit upregulated TRPV1 expression and function, and pharmacological inhibition of TRPV1 attenuates disease symptoms (24).

Activated sensory neurons engage in bidirectional signaling. Centrally, their terminals propagate action potentials to the trigeminal spinal nucleus—the first relay station of the nose-brain axis (3). Notably, this enhanced afferent input to the spinal trigeminal nucleus does not merely relay sensory information; it also stimulates the superior salivatory nucleus, driving a parasympathetic reflex that promotes vasodilation and glandular hypersecretion (33). This trigemino-parasympathetic reflex accounts for the nasal congestion and rhinorrhea characteristic of AR, and represents a direct link between peripheral sensory sensitization and brainstem-mediated autonomic effector responses (21, 34). Peripherally, they release neuropeptides (substance P and CGRP) stored in dense-core vesicles via antidromic axonal transport, a process dependent on calcium influx and SNARE-mediated exocytosis (29, 30). In the sensitized state, heightened responsiveness to inflammatory mediators and increased membrane expression of TRPV1/TRPA1 amplify calcium influx and neuropeptide release (4, 31). These neuropeptides are potent vasodilators that induce plasma extravasation, glandular secretion, and chemotaxis of immune cells (4).

In AR, this neurogenic inflammation synergizes with immune-mediated inflammation to establish a self-amplifying positive feedback loop: inflammatory mediators activate sensory neurons to release neuropeptides, which further exacerbate local inflammation; the intensified inflammatory milieu in turn sustains neuronal sensitization and neuropeptide release (15). This loop perpetuates peripheral sensitization and ensures continuous transmission of aberrant signals to the CNS (15, 32).

## 4 Signal encoding and transmission along primary afferent pathways

The trigeminal nerve fulfills a dual role in allergic rhinitis (AR): it transmits inflammatory signals from the nasal cavity to the CNS (acting as a “messenger”) and simultaneously amplifies peripheral inflammation through neuropeptide release (acting as a “participant”) (40). As the fifth cranial nerve, its ophthalmic and maxillary divisions innervate the nasal and sinus mucosa, constituting the principal afferent pathway for nasal sensation (33, 34). The cell bodies of trigeminal neurons reside predominantly within the trigeminal ganglion (TG), a major sensory ganglion located in the middle cranial fossa (35). The trigeminal ganglion (TG) exhibits considerable neuronal heterogeneity in molecular phenotype and functional properties, reflected by differential expression of neuropeptides, ion channels, and receptors across neuronal subpopulations (36). Classical neuropeptides (SP, CGRP) are predominantly expressed in a subset of peptidergic nociceptive neurons, whereas TRPV1 channels are highly enriched in, but not exclusive to, these peptidergic neurons, and are also expressed in certain non-peptidergic nociceptor subpopulations (37, 38). Multiple functionally distinct neuronal subpopulations have been identified within the trigeminal ganglion (TG), including peptidergic nociceptors (PEP1/2), non-peptidergic nociceptors (NP1/2), C-fiber low-threshold mechanoreceptors, and myelinated neurons (36).

Central projections from the TG extend to the brainstem, terminating primarily in the trigeminal spinal nucleus and the principal sensory nucleus (39). These nuclei serve as critical relay stations for the initial integration of nasal sensory information. Following processing, second-order neurons relay this information to the ventral posteromedial (VPM) thalamus and ultimately to the somatosensory cortex, giving rise to the conscious perception of odor, pain, or discomfort (34). This multi-level pathway provides the anatomical substrate for information transmission along the nose-brain axis.

## 5 Signal reception and initial integration in primary brainstem centers

### 5.1 The trigeminal spinal nucleus as the primary brainstem gateway

The trigeminal spinal nucleus is the first brainstem relay station for nasal sensory input, receiving direct projections from the trigeminal ganglion (TG) (40). This anatomical position designates it as the critical primary gateway through which nasally derived sensory signals—including itch, pain, and discomfort—enter the CNS (40). In AR, neurons within this nucleus exhibit pronounced functional and structural plasticity, marking the onset of central sensitization. Peripheral inflammatory stimulation directly enhances neuronal activity in the trigeminal spinal nucleus, as evidenced by the upregulation of c-Fos in nociceptive neurons (3). Such alterations reflect neuronal functional remodeling, potentially accompanied by long-term potentiation (LTP) of synaptic efficacy, which lowers activation thresholds and amplifies incoming signals (41, 42). As a critical nexus bridging the peripheral and central compartments of the nose-brain axis, sensitization of the trigeminal spinal nucleus links peripheral nasal inflammation to functional changes within the CNS, potentially contributing to comorbidities such as headache (43).

### 5.2 Thalamic relay dysfunction and aberrant signal gating

Processed sensory information ascends via spinothalamic and trigeminothalamic tracts to specific thalamic nuclei, notably the ventral posteromedial nucleus (44). The thalamus, a critical relay station for sensory information en route to the cortex, may undergo substantial functional reorganization in the context of AR-associated central sensitization (45). Such reorganization may manifest as increased spontaneous activity of thalamic neurons and dysregulated modulation of evoked responses, leading to impaired gating of incoming nasal signals (45). Under these conditions, the precise “gate control” mechanism fails, allowing non-noxious or low-intensity nasal stimuli to be aberrantly amplified and relayed rostrally, directly contributing to central sensitization (4, 22).

The thalamus also serves as a key hub interconnecting the cerebellum, basal ganglia, and cortical networks (4). Rather than functioning as a passive relay station, it operates as a dynamic center for information processing and distribution, routing amplified nasal sensory information to somatosensory cortices for localization and discrimination, and to limbic structures (amygdala, anterior cingulate cortex) for emotional and cognitive appraisal (46, 47).

## 6 Remodeling of higher-order brain regions and behavioral correlates

### 6.1 The limbic system and emotional-cognitive processing

Beyond generating aberrant sensory input, peripheral nasal inflammation in AR disrupts sleep architecture through both mechanical and neuroimmune pathways (8, 104).Nasal congestion predisposes to REM-related sleep-disordered breathing and respiratory effort-related arousals that fragment sleep (104).Concurrently, inflammatory mediators may directly modulate central sleep-regulatory centers (7).The resulting REM disruption and sleep fragmentation impair emotional regulation and cognitive performance (55, 65), thus serving as a mechanistic link between peripheral nasal inflammation and limbic dysfunction.

Within this context, the limbic system—which includes the amygdala, hippocampus, and anterior cingulate cortex (ACC)—emerges as a core network responsible for emotion, memory, and cognitive processing (48, 49).These regions are interconnected through complex neural circuits that integrate sensory input, affective experience, and cognitive appraisal to generate coordinated behavioral and physiological responses (48, 50). The amygdala rapidly evaluates the emotional salience of stimuli, whereas the ACC is involved in conflict monitoring, attentional allocation, and the subjective experience of discomfort (49, 51).

Upon allergen challenge, aberrant sensory signals reach the limbic system via thalamic relays. Functional neuroimaging studies have demonstrated that limbic structures exhibit abnormally enhanced activity during the processing of negative emotional stimuli (52–54). This limbic hyperactivation is closely associated with negative emotional states in AR patients. Heightened amygdala reactivity correlates with anxiety, irritability, and catastrophic cognitive appraisals of symptoms (55, 56). Concurrently, abnormal ACC activity is linked to an attentional bias toward uncomfortable sensations (10, 57). Enhanced ACC activity may drive sustained attentional capture and fixation on nasal discomfort, impairing attentional disengagement and amplifying subjective distress (58). This limbic system-mediated alteration in emotional and cognitive processing constitutes a core component of central sensitization in AR, explaining why patients with comparable objective inflammation exhibit marked differences in symptom perception and associated distress (59, 60).

### 6.2 Cortical sensory remapping and cognitive dysfunction

Prolonged aberrant sensory input may drive cortical remapping—a functional reorganization of the sensory cortex. Chronic nasal inflammation may similarly induce reorganization of the nasal representation zones within the primary and secondary somatosensory cortices (61, 62). Such central remodeling may underlie phantom olfactory perceptions or persistent nasal discomfort even after objective signs of inflammation have resolved (63, 64).

Higher-order cognitive regions, including the prefrontal cortex (PFC), are also susceptible to disruption. Neuroinflammation and sustained aberrant sensory input may collectively impair their normal function. Neuroinflammatory cytokines, such as IL-6, play a critical role in mediating pain-associated cognitive impairment (65, 66). In AR, nasal microbiota dysbiosis may influence the central nervous system (CNS) via olfactory neural pathways, systemic immune signaling, and other routes, leading to blood–brain barrier (BBB) dysfunction and microglial activation, which in turn may perturb PFC and other brain regions (7, 67). Such interference may manifest as slowed thinking, impaired concentration, and memory decline—a symptom complex frequently referred to as “brain fog” (68, 69).

Notably, the central remodeling induced by AR exhibits neuroplasticity. This plasticity not only underlies symptom persistence but also provides a theoretical rationale for reversing sensitization through targeted interventions. Behavioral interventions such as cognitive behavioral therapy (CBT) operate, in part, by modifying cognitive and behavioral patterns to reshape brain function and structure (70, 71). Working memory training has been shown to enhance cognitive performance and improve neural efficiency (72, 73). Thus, elucidating the plasticity mechanisms underlying central sensitization in allergic rhinitis (AR) may open new avenues for non-pharmacological management strategies.

## 7 Central neuroimmune interactions and neuroinflammation

Peripheral inflammatory signals gain access to the central nervous system (CNS) via olfactory, trigeminal, or humoral routes, triggering the activation of resident microglia and astrocytes (5) (**Table 1**). In AR models, both glial cell types are markedly activated in the trigeminal spinal subnucleus caudalis (TNC), providing a cellular foundation for central sensitization (3, 74). Microglial activation typically precedes and promotes astrocyte activation (75).

**Table 1:** Key molecular mediators involved in the nose-brain axis–driven central sensitization and neuroinflammation in allergic rhinitis.

Mediator	Source/expression	Primary target	Pathological effect in central sensitization	References
TRPV1/ TRPA1	Trigeminal sensory neurons	Ion channels on sensory nerve endings	Lowered activation threshold; enhanced calcium influx; neuropeptide release	(24, 26)
Substance P/CGRP	Peptidergic nociceptors	Vascular endothelium; immune cells; glial cells	Vasodilation; plasma extravasation; neurogenic inflammation; glial activation	(15, 85)
Glutamate	Primary afferent neurons; central neurons	NMDAR, AMPAR on postsynaptic neurons	Excitotoxicity; LTP-like synaptic strengthening; central sensitization consolidation	(86, 87)
IL-1β, TNF-α, IL-6	Microglia; astrocytes	Neurons; glial cells	Neuronal hyperexcitability; synaptic impairment; neuroinflammation propagation	(88, 89)
Cytokines (e.g., IL-6, IL-1β)	Central cytokine network	Neuronal receptors; neurotransmitter metabolism	Network dysregulation; IDO activation; behavioral and affective symptoms	(90, 91)
NLRP3 inflammasome	Microglia in TNC	Pro-caspase-1; IL-1β precursor	IL-1β production; proinflammatory microglial polarization	(3, 79, 92)

Abbreviations are provided in the main Abbreviations list. This table summarizes principal molecular hubs, their cellular sources, primary targets, and the pathological consequences that collectively drive central sensitization and neuroinflammation in AR.

Activated microglia polarize toward a proinflammatory (M1-like) phenotype, releasing TNF-α and IL-1β, which directly lower neuronal excitation thresholds (76, 77). In AR, microglia within the trigeminal spinal subnucleus caudalis (TNC) also activate the NLRP3 inflammasome, thereby increasing IL-1β production (78, 79). Microglia can further transmit inflammatory signals via exosomes, driving astrocytes toward a neurotoxic phenotype (75, 80). Activated astrocytes sustain neuroinflammation by releasing chemokines, glutamate, and ATP, directly modulating neuronal activity (81). Microglial exosomes can induce astrocytic A1 polarization, contributing to neuronal injury and synaptic dysfunction (82, 83). Moreover, microglia can prune astrocytic processes, thereby reducing glutamate clearance and culminating in neuronal hyperexcitability (84). Thus, astrocyte activation serves as a critical bridge linking neuroinflammation to neuronal dysfunction and central sensitization (**Figure 3**).

## 8 Imbalance of neurotransmitter systems: glutamatergic hyperexcitation and descending dysregulation

### 8.1 Hyperexcitation of the glutamatergic system

Glutamate is the principal excitatory neurotransmitter in the CNS, and aberrant activation of its signaling pathways is a key mechanism underlying central sensitization (86, 93). In AR, persistent peripheral inflammation induces excessive glutamate release from primary afferents into trigeminal spinal subnucleus caudalis (TNC) in the brainstem (3, 86). This excessive release disrupts the excitatory–inhibitory balance and initiates central sensitization.

Glutamate acts through ionotropic receptors, and upregulation of NMDARs and AMPARs is central to central sensitization (94). In AR, repeated allergen exposure upregulates these receptors and alters subunit composition, enhancing glutamate sensitivity and Ca²^+^ influx (10, 94). Elevated Ca²^+^ activates CaMKII, PKC, and ERK, which phosphorylate receptor subunits to increase open probability and promote receptor insertion into the postsynaptic membrane (95, 96). These kinases also translocate to the nucleus to phosphorylate CREB, inducing immediate early and plasticity-related genes (e.g., c-Fos) (3). This self-reinforcing cycle culminates in LTP-like synaptic strengthening, lowering neuronal thresholds, amplifying nasal signal responses, and consolidating central sensitization (4, 53)—clinically manifesting as nasal hyperresponsiveness and associated headache.

### 8.2 Dysregulation of descending modulatory systems

The brain possesses endogenous descending modulatory circuits—including the periaqueductal gray (PAG), rostral ventromedial medulla (RVM), and noradrenergic/serotonergic pathways—that normally exert “gate control” over ascending afferent signals (18, 97). In allergic rhinitis (AR), descending inhibitory function is attenuated, whereas facilitatory influences originating from limbic structures (amygdala, ACC) are enhanced (4, 98).

The amygdala, a key hub integrating nociceptive and affective information, contributes to neuronal sensitization (18, 99). Serotonergic signaling can exert dual effects: 5-HT_1_F receptors mediate analgesic effects and inhibit CGRP release (100), but dysfunction within prefrontal–limbic circuitry impairs emotional–cognitive appraisal and augments descending facilitation (10, 101). Consequently, weak nasal stimuli may be perceived as intense itching or discomfort—manifesting as central sensitization symptoms, including reactivity to non-allergic triggers and persistent nasal paresthesia (4, 31, 102). Restoring the balance between inhibition and facilitation represents an important therapeutic strategy (90, 103).

## 9 Potential novel therapeutic targets targeting the nasal-brain axis and considerations for conventional medications

Conventional pharmacotherapy for AR acts peripherally to suppress local inflammation, reduce histamine release, alleviate mucosal edema, and diminish inflammatory cell infiltration, thereby indirectly modulating peripheral afferent signals along the nose-brain axis (104, 105). However, once central sensitization is established, these medications often demonstrate limited efficacy because they primarily target peripheral inflammation (104, 106). Central sensitization entails neuroplastic changes, glial activation, and neurotransmitter dysregulation—alterations that extend beyond the scope of conventional anti-inflammatory agents (3) (**Table 2**). Therefore, although standard medications remain first-line therapy, their efficacy may be suboptimal in patients with refractory AR who present with chronic pain-like symptoms (e.g., persistent nasal irritation, headache) or emotional comorbidities.

**Table 2:** Emerging therapeutic strategies targeting the nose-brain axis, neuroinflammation, and central sensitization in allergic rhinitis.

Intervention category	Strategy	Target/mechanism	Therapeutic rationale	References
Peripheral nerve modulation	TRPV1 antagonists	TRPV1 channels on trigeminal neurons; block calcium influx; reduce neuropeptide release	Suppress peripheral sensitization and neurogenic inflammation at the first gateway	(24, 107)
Central neuroinflammation modulation,	Minocycline	Microglial activation; inhibit proinflammatory polarization; reduce TNF-α, IL-1β	Attenuate central neuroinflammation and glial-mediated sensitization	(3, 108)
Central neuroinflammation modulation	Microglial/astrocytic signaling inhibitors (TLR4/NF-κB, JAK/STAT)	Glia-specific signaling pathways; suppress proinflammatory phenotype	Targeted inhibition of central immune activation	(109–111)
Neurotransmitter balance restoration	Low-dose ketamine	NMDA receptor antagonism; restore synaptic homeostasis; block calcium influx	Reverse central sensitization; address comorbid mood symptoms	(112, 113)
Neurotransmitter balance restoration	SNRIs (e.g., duloxetine)	Serotonin/norepinephrine reuptake inhibition; enhance descending inhibitory pathways	Strengthen gate control; suppress excessive afferent signals	(114, 115)
Neuromodulation	Transcranial magnetic stimulation	Prefrontal-limbic circuits; modulate cortical excitability	Reshape aberrant neural circuits; restore inhibitory–facilitatory balance	(116)
Neuromodulation	Transcutaneous electrical nerve stimulation (TENS)	Trigeminal nerve branches; modulate afferent impulses	Reset brainstem neuronal excitability; reduce nasal hypersensitivity	(117, 118)
Psychobehavioral intervention	Cognitive behavioral therapy	Limbic system; cognitive appraisal; emotional processing	Modify maladaptive cognitive patterns; disrupt symptom–anxiety cycle	(119, 120)
Drug delivery optimization	Intranasal drug delivery	Nose-brain axis pathway; bypass blood–brain barrier	Enhance intracranial bioavailability; reduce systemic side effects	(121, 122)

Abbreviations are provided in the main Abbreviations list. The strategies listed represent a spectrum from established clinical applications to emerging preclinical concepts; their inclusion reflects mechanistic plausibility and therapeutic potential rather than current regulatory approval status for AR.

While conventional medications may indirectly attenuate aberrant immune signals transmitted to the CNS by suppressing peripheral inflammation, the precise molecular and circuit-level mechanisms through which they influence central sensitization via the nose-brain axis require further elucidation (105). Future drug development should move beyond single-target receptor blockade toward multi-target, temporally precise interventions aimed at restoring homeostatic balance within the neuroimmune system.

## 10 Future research challenges and directions

Although our understanding of the role of the nose-brain axis in AR-associated central sensitization continues to advance, several key challenges remain. First, the primary challenge lies in accurately distinguishing between peripheral inflammatory components and central sensitization components in AR (3, 4). Multimodal approaches are required to objectively quantify CNS changes. Neuroimaging techniques such as functional magnetic resonance imaging (fMRI) and magnetic resonance spectroscopy (MRS) can monitor central metabolic status; one ¹H-MRS study demonstrated that exposure to allergens and PM2.5 altered brainstem glutamine and glutamate levels, correlating with sensitization of trigeminal TRPV1^+^ neurons (86). Furthermore, cerebrospinal fluid (CSF) or blood markers of glial activation (e.g., GFAP, NfL) may provide valuable clues for assessing AR-associated neuroinflammation (123).

Second, future research should aim to establish causal evidence linking specific nasal inflammatory mediators (e.g., IL-4, IL-13) to distinct central sensitization phenotypes. Although a recent study by Lv and colleagues (2025) provided direct evidence for IL-4-mediated NLRP3 inflammasome activation in microglia (3), independent replication and extension to other relevant cytokines are warranted.

Third, clinical trials explicitly targeting central sensitization in AR are urgently needed. Subgroups of AR patients exhibiting clear biomarkers of central sensitization could be identified for individualized interventions. Non-pharmacological approaches—including cognitive behavioral therapy (CBT), transcranial magnetic stimulation (TMS), and transcutaneous electrical nerve stimulation (TENS)—warrant systematic evaluation (119, 124, 125). Acupuncture may also modulate the hypothalamic–pituitary–adrenal (HPA) axis (126, 127), suggesting broader neuromodulatory potential. In addition, prefrontal–subcortical imbalance may constitute a critical neural basis that bridges allergic inflammation with affective and somatic manifestations (128, 129). Whether PFC hypoactivity represents a primary driver or a secondary consequence of AR-related central sensitization remains unclear, and future neuroimaging studies combining resting-state functional connectivity with cognitive challenge tasks may help clarify this issue. Finally, given the bidirectional relationship between AR and psychological stress, psychobehavioral interventions may help disrupt the “symptom–anxiety–symptom exacerbation” cycle.

## 11 Conclusion

Allergic rhinitis is no longer merely a localized mucosal inflammatory disorder; rather, it is a systemic condition involving hierarchical neuroimmune regulation via the nose-brain axis. Central sensitization—driven by persistent peripheral inflammatory signaling, trigeminal afferent transmission, neuroinflammation, glial activation, and neurotransmitter imbalance—underlies the chronicity, refractoriness, and neuropsychiatric comorbidities frequently observed in AR. This review has systematically delineated the multi-level mechanisms of neuroimmune crosstalk within the nose-brain axis, spanning from peripheral initiation to central integration and neuromodulatory imbalance. We have also highlighted promising therapeutic strategies that target neuroinflammation, glial function, and neural circuits. This integrated framework advances the mechanistic understanding of AR and provides novel targets for developing more effective interventions for patients with refractory AR complicated by central sensitization and neurobehavioral comorbidities.
